# Invasive fusariosis after CD19 chimeric antigen receptor T-cell therapy

**DOI:** 10.1128/asmcr.00117-24

**Published:** 2025-05-08

**Authors:** Rita Wilson Dib, Annoir Shayya, Emily A. Siegrist, Maria Alkozah, Brian Scott, José Henao-Cordero, Cindy McCloskey, Melanie Speckman, Jeffrey D. McBride, Jennifer Holter-Chakrabarty, Joseph Sassine

**Affiliations:** 1 Infectious Diseases Section, Department of Medicine, The University of Oklahoma Health Sciences Center 6186, Oklahoma City, Oklahoma, USA; 2 Department of Hematology & Medical Oncology, Lebanese American University-Rizk Hospital 502749, Beirut, Lebanon; 3 Department of Microbiology, Oklahoma University Health Sciences Center 6186 , Oklahoma City, Oklahoma, USA; 4 Department of Pathology, University of Oklahoma Health Sciences Center 6186, Oklahoma City, Oklahoma, USA; 5 Department of Dermatology, University of Oklahoma Health Sciences Center 6186, Oklahoma City, Oklahoma, USA; 6 Hematology-Oncology Section, Department of Medicine, University of Oklahoma Health Sciences Center 6186, Oklahoma City, Oklahoma, USA; Pattern Bioscience, Austin, Texas, USA

**Keywords:** Fusarium, CAR-T, skin nodules, case report

## Abstract

**Background:**

Invasive fusariosis is rarely reported post-chimeric antigen receptor T-cell (CAR-T) therapy. We herein present a case of cutaneous invasive *Fusarium* infection and provide a compilation of similar cases documented in the existing literature.

**Case Summary:**

A 61-year-old woman with relapsed refractory diffuse large B-cell lymphoma and secondary hemophagocytic lymphohistiocytosis received CD19-CAR-T therapy. She developed grade 1 cytokine release syndrome (CRS) and grade 3 immune effector cell-associated neurotoxicity syndrome (ICANS), requiring dexamethasone and anakinra. Twenty-five days after CAR-T, she developed bilateral proximal thigh nodular lesions. Skin biopsy revealed hyphal structures with hyphal structures, and culture revealed *Fusarium* species. Treatment with liposomal amphotericin B, voriconazole, and terbinafine followed by voriconazole and terbinafine led to clinical improvement.

**Conclusion:**

Though rare, healthcare providers should maintain an index of suspicion for *Fusarium* infections in recipients of cellular therapies with risk factors.

## INTRODUCTION

Invasive fusariosis is a rare entity that primarily affects patients with hematological malignancies and recipients of hematopoietic cell transplants. Cases of mold infections in chimeric antigen receptor T-cell (CAR-T) therapy recipients have seldom been reported (1–6). We herein present this case of invasive fusariosis managed with combination antifungal therapy. Additionally, we provide a succinct review of cases reported in existing literature.

## CASE PRESENTATION

The patient is a 61-year-old woman with relapsed, refractory diffuse large B-cell lymphoma and secondary hemophagocytic lymphohistiocytosis (HLH). She received six cycles of R-EPOCH with persistent residual disease without additional specific therapies for HLH. Furthermore, she developed central nervous system disease, diagnosed while receiving fludarabine and cyclophosphamide lymphodepletion in preparation for planned CD19 CAR-T therapy with axicabtagene ciloleucel. Hence, she received intrathecal cytarabine, hydrocortisone, and methotrexate simultaneously. Prior to the CAR-T infusion, she had CMV DNAemia reaching 2,270 IU/mL (reference range: 34.5–4,000,000) without evidence of end-organ disease. She was treated with foscarnet induction, followed by letermovir for secondary prophylaxis. She developed severe neutropenia (<500 cells/uL) for 24 consecutive days. She was receiving micafungin 50 mg daily prophylaxis and then switched to posaconazole 300 mg once daily on day 1 after the CD19 CAR-T infusion per institutional protocol.

**Fig 1:**
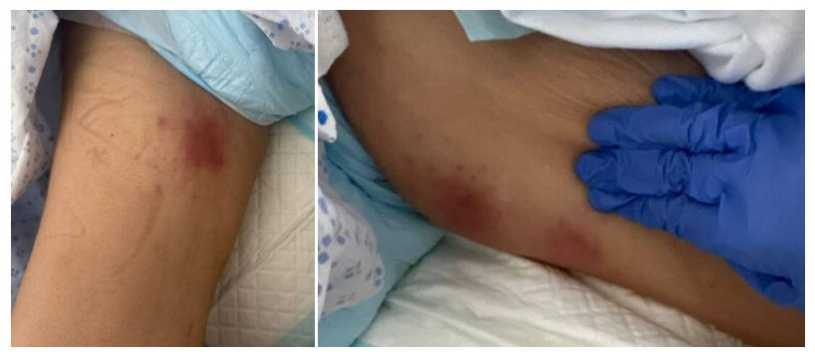
Skin nodules on bilateral inner thighs.

She experienced grade 1 cytokine release syndrome (CRS) and grade 3 immune effector cell-associated neurotoxicity syndrome (ICANS), which were treated with dexamethasone/methylprednisolone (equivalent to 4.4 g prednisone) for 11 days and anakinra 100 mg administered on an every 6 hour basis for 5 days. She also received intravenous immunoglobulins for hypogammaglobulinemia. Twenty-five days after her CAR-T therapy, she developed bilateral nodular erythematous and tender lesions of the medial proximal thighs (Fig. 1).

**Fig 2:**
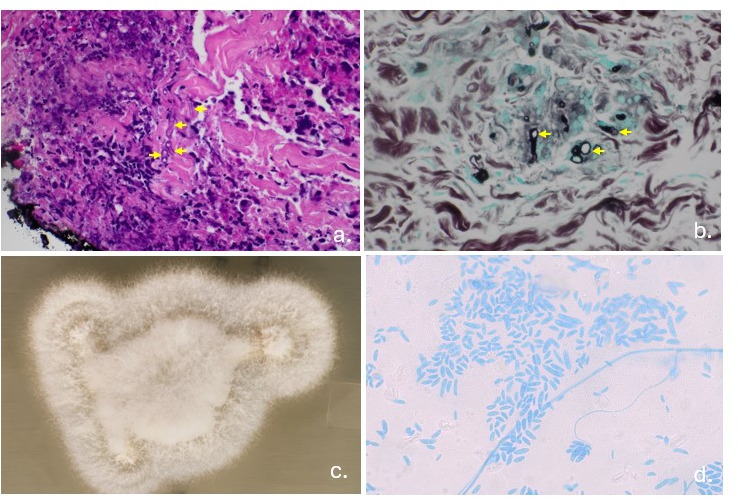
Histopathologic and microbiologic findings. (a) Subcutaneous hyphal structures on H&E stain. (b) Fungal hyphae visible on GMS stain. (c) Gross fungal growth on inhibitory mold agar. (d) Hyaline, septate hyphae with characteristic curved macroconidia visualized under the microscope at ×400 magnification.

**Fig 3:**
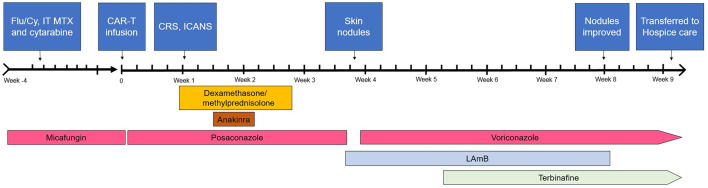
Timeline of clinical events in the presented case. Flu, fludarabine; Cy, cyclophosphamide; IT, intrathecal; MTX, methotrexate; CRS, cytokine release syndrome; ICANS, immune effector cell-associated neurotoxicity syndrome; LAmB, liposomal amphotericin B.

She had recovered her neutrophil count for 2 weeks by that time. Skin biopsy revealed hyphal structures concerning for fungal elements on Grocott methenamine silver and periodic acid Schiff-fungal stains (Fig. 2). Cultures revealed *Fusarium* species (susceptibility being 1 mcg/mL for amphotericin B, >16mcg/mL for posaconazole, >16mcg/mL for voriconazole, and >16mcg/mL for isavuconazole). CT scans of the sinuses and chest and blood cultures were unrevealing. The serum aspergillus galactomannan and (1,3)-beta-D-glucan were negative, and the posaconazole level at the time was 2.2 μg/mL. Treatment included liposomal amphotericin B at 5 mg/kg every 24 hours for 6 weeks, along with oral voriconazole (dose adjusted multiple times to achieve therapeutic levels). Subsequently, oral terbinafine 250 mg twice daily was added to the regimen, resulting in significant clinical improvement and resolution of the lesions. Due to the overall grim prognosis and severe deconditioning endured in the immediate post-CAR-T period, the patient eventually decided to pursue hospice care (Fig. 3).

## DISCUSSION

CAR-T therapy has rapidly evolved over the past decade, becoming a cornerstone in hematologic malignancy immunotherapy. While effective, these drugs can be associated with unintended consequences of toxicities and immune deficiencies. In the early post-conditioning period, myelosuppression commonly occurs and is further perpetuated by CRS or ICANS and their corresponding therapies (6, 7). The CD19-targeted CAR-T therapy is associated with prolonged B-cell aplasia, and BCMA-targeting therapy can further lead to plasma cell depletion.

The incidence of invasive mold infections in recipients of cellular therapy is reported to range between 0% and 6% (7–9) with more data available on infections in CD19 CAR-T recipients. *Fusarium* infections are rarely described in these patients. Among 85 patients at a US center who underwent CD19 CAR-T therapy, one case (1.2%) of *Fusarium* was reported in the early posttreatment period (within 30 days) (1). In another cohort of 50 patients, one case (2%) occurred between days 30 and 90 (2). In a French cohort of 1,144 CAR-T recipients, which reported 32 cases (3%) of mold infections, only 1 was caused by *Fusarium* species and was recovered from the blood during the first 30 days post-CAR-T (3). In a cohort of 92 patients from China, with two cases of fungal infections, one was attributed to invasive fusariosis (6).

The rest of the cases in the literature are presented as independent case reports (4, 5) (Table 1).

**TABLE 1:** Summary of cases of invasive fusariosis post-CAR-T therapy^*a*^

Source	Primary malignancy	Time to infection	Toxicity	Immunosuppressive therapies	Type of infection	Antifungal prophylaxis	Treatment; outcome
(1) Logue et al.	LBCL	Day 0–30	ICANS	Steroids	Disseminated fusariosis	Fluconazole then micafungin	–, death
(2) Walker et al.	DLBCL	Day 55	CRS	Treatment for CRS not specified	Blood	Isavuconazole	–
(3) Bouvier et al.	–	–	CRS	–	Blood	–	–
(4) Chesdachai et al.	DLBCL	1 month	CRS and HLH	Tocilizumab High-dose steroids	Invasive sinusitis	Fluconazole	LamB, voriconazole, –
(5) Haider et al.	ALL	Day 22	CRS	Tocilizumab	Skin and sinuses	Fluconazole then posaconazole	LamB, voriconazole, terbinafine, amphotericin B nasal irrigations; improved
(6) Zhu et al.	NHL	Day 52	–	–	–	–	–
Wilson Dib et al.	DLBCL	Day 25	CRS, ICANS	Steroids, anakinra	Skin	Posaconazole	LamB, voriconazole, terbinafine; death

^*a*^ LBCL, diffuse large B-cell lymphoma; ALL, acute lymphocytic leukemia; NHL, non-Hodgkin’s lymphoma; CRS, cytokine release syndrome; ICANS, immune effector cell-associated neurotoxicity syndrome; LamB, liposomal amphotericin B; –, not specified.

The onset of mold infections after CAR-T therapy appears to follow a similar timeline to that of allogeneic hematopoietic cell transplant recipients, often occurring within the first 30 days of cellular therapy (10) but has also been reported after 30 days (11) in patients with and without mold active prophylaxis (11). Host-related risk factors for mold infections in recipients of cellular therapy vary by underlying disease and prior treatments. Patients with leukemia, relapsed refractory lymphoma, and those with prior allogeneic hematopoietic cell transplants, particularly if on active GVHD therapy, are at higher risk for developing invasive fungal infections (6, 11). Post CAR-T, those with prolonged neutropenia, immunosuppressive treatments used for CAR-T-associated toxicities (CRS/ICANS) such as steroids and interleukin inhibitors, delayed cytopenias, and T-cell deficiencies further increase the risk of acquiring invasive fungal infections (10, 11). Many of the stated factors were present in our case, warranting mold-active prophylaxis. Additionally, we hypothesize that the patient’s body habitus, immobility, and ongoing thigh-to-thigh contact facilitated the development of the infection with bilateral medial symmetric distribution.

Fusariosis in this patient population commonly manifests as disseminated disease with refractory fevers, and pulmonary, sinus, and skin involvement in the form of multiple skin lesions (12, 13). Fusarium species can also be detected in blood cultures (12, 13). While Aspergillus galactomannan and (1,3)-beta-D-glucan can be positive in some cases of fusariosis (13), they were both negative in our patient.

Treatment of invasive fusariosis is not well described due to the lack of randomized trials and the lack of correlation between *in vitro* susceptibilities and clinical outcomes. Its low incidence limits studies of this infection. Localized infections are likely to benefit from surgical debridement (12). Combination therapy with amphotericin B and voriconazole, or voriconazole plus terbinafine, or amphotericin B with terbinafine has been used with inconsistent results (13, 14). That may be because some species, particularly those belonging to the solani complex, exhibit higher MICs to azoles and amphotericin B (13). In patients with hematological malignancy, the combination of azoles and terbinafine has been suggested to be effective for localized skin infections (15).

Preventive measures primarily focus on averting exposure and early identification of risk factors, including onychomycosis, intertrigo, or areas of skin breakdown with subsequent contact with tap water. *Fusarium* species are frequently recovered from hospital water systems worldwide. Chemoprophylaxis has not been shown to decrease the overall incidence of *Fusarium* infections, with breakthroughs being more prevalent in patients receiving standard posaconazole prophylaxis (16). Benefit was suggested, however, in a small subset of patients with high-risk hematologic malignancies who had concomitant baseline superficial skin lesions and studies suggestive of *Fusarium* species (17).

In conclusion, though rare, healthcare providers should maintain an index of suspicion for *Fusarium* infections in recipients of cellular therapies with known risk factors. Treatment should involve a combination of antifungal therapy and debridement when applicable.
